# Quantitative Evaluation of COVID-19 Pneumonia CT Using AI Analysis—Feasibility and Differentiation from Other Common Pneumonia Forms

**DOI:** 10.3390/diagnostics13122129

**Published:** 2023-06-20

**Authors:** Una Ebong, Susanne Martina Büttner, Stefan A. Schmidt, Franziska Flack, Patrick Korf, Lynn Peters, Beate Grüner, Steffen Stenger, Thomas Stamminger, Hans Kestler, Meinrad Beer, Christopher Kloth

**Affiliations:** 1 Department of Diagnostic and Interventional Radiology, Ulm University Medical Center, Albert-Einstein-Allee 23, 89081 Ulm, Germanymeinrad.beer@uniklinik-ulm.de (M.B.); 2 Scientific Collaborations Siemens Healthcare GmbH Erlangen, 91052 Erlangen, Germany; 3 Division of Infectious Diseases, University Hospital and Medical Centre of Ulm, 89081 Ulm, Germany; 4 Institute of Medical Microbiology and Hygiene, Ulm University Medical Center, 89081 Ulm, Germany; 5 Institute of Virology, Ulm University Medical Center, 89081 Ulm, Germany; 6 Institute for Medical Systems Biology, Ulm University, 89081 Ulm, Germany

**Keywords:** COVID-19 pneumonia, CT, automated segmentation and quantification

## Abstract

**PURPOSE:** To implement the technical feasibility of an AI-based software prototype optimized for the detection of COVID-19 pneumonia in CT datasets of the lung and the differentiation between other etiologies of pneumonia. **METHODS:** This single-center retrospective case–control-study consecutively yielded 144 patients (58 female, mean age 57.72 ± 18.25 y) with CT datasets of the lung. Subgroups including confirmed bacterial (*n* = 24, 16.6%), viral (*n* = 52, 36.1%), or fungal (*n* = 25, 16.6%) pneumonia and (*n* = 43, 30.7%) patients without detected pneumonia (comparison group) were evaluated using the AI-based *Pneumonia Analysis prototype*. Scoring (extent, etiology) was compared to reader assessment. **RESULTS:** The software achieved an optimal sensitivity of 80.8% with a specificity of 50% for the detection of COVID-19; however, the human radiologist achieved optimal sensitivity of 80.8% and a specificity of 97.2%. The mean postprocessing time was 7.61 ± 4.22 min. The use of a contrast agent did not influence the results of the software (*p* = 0.81). The mean evaluated COVID-19 probability is 0.80 ± 0.36 significantly higher in COVID-19 patients than in patients with fungal pneumonia (*p* < 0.05) and bacterial pneumonia (*p* < 0.001). The mean percentage of opacity (PO) and percentage of high opacity (PHO ≥ −200 HU) were significantly higher in COVID-19 patients than in healthy patients. However, the total mean HU in COVID-19 patients was −679.57 ± 112.72, which is significantly higher than in the healthy control group (*p* < 0.001). **CONCLUSION:** The detection and quantification of pneumonia beyond the primarily trained COVID-19 datasets is possible and shows comparable results for COVID-19 pneumonia to an experienced reader. The advantages are the fast, automated segmentation and quantification of the pneumonia foci.

## 1. Introduction

Since December 2019, the world has been confronted with a novel coronavirus (nCoV), termed SARS-CoV-2, announced by the World Health Organization (WHO) as being responsible for the outbreak of COVID-19 [1,2,3,4]. It first emerged in Wuhan, China [4], and continued to spread all over the globe, taking almost 6 million lives [5]. The pandemic caused by the virus naturally represented a major challenge for healthcare systems worldwide. Patients infected with coronavirus disease 2019 (COVID-19) suffer from pneumonia-like symptoms, mainly including fever, cough, shortness of breath, and fatigue [6]. The clinical presentation, therefore, resembled previous outbreaks of other coronaviruses, such as severe acute respiratory distress syndrome (SARS-1) and Middle East respiratory virus (MERS) [7]. The incidence of SARS-CoV in 2002 and 2003 and MERS-CoV in 2012 has already shown the potential for the transmission of newly emerging CoVs from animal to human and person to person [1]. In total, seven human coronaviruses (HCoVs) have now been discovered [1]. COVID-19 was the third highly epidemic disease to be detected, with a lower mortality rate than SARS and MERS, depending on the country [1]. The higher transmissibility and varied clinical manifestations of COVID-19 could be a result of the diversity in the biology and genome structure compared to the other human coronaviruses [1,8,9]. Nevertheless, substantial mortality and residual symptoms can occur.

These may present as breathlessness and lowered quality-of-life-measures [7,10].

In the absence of specific therapeutic drugs or vaccines for COVID-19 at the beginning of the disease and limited possibilities of PCR testing, lung imaging was of particular importance [11]. In this phase of the pandemic, medical imaging was considered to be a first-line investigation tool [12,13].

The chest X-ray, as well as computed tomography (CT), have been proven to be accurate tools for detecting COVID-19 [14,15,16]. However, image interpretation puts a great burden on radiologists, providing a growing workload during times of the pandemic. In this clinical context, the approach of automated software for giving proof of COVID-19 pneumonia with an output of a probability score was tested. 

Feasible studies on the differentiation of similar pneumonia based on CT parameters had existed before the pandemic started [17].

Chabi ML et al. have already shown, with a small cohort, the extent of lung infiltration assessed on CT scan by using software independently, which definitely predicts the risk of clinical deterioration or in-hospital death [18].

Artificial intelligence (AI) using deep learning technology has demonstrated great success in the medical imaging domain due to its high capability of feature extraction [19,20]. Artificial intelligence has shown promising results in both the diagnosis and prognosis of COVID-19 [21]; the applications during the pandemic were manifold [22]. 

The first approaches deal with the identification and successively the severity quantification of COVID-19 in lung CT [13]. Currently, numerous applications of AI, in different forms, are proposed to facilitate several clinical tasks in the management of COVID-19 [23].

Specifically, deep learning was applied to detect and differentiate pneumonia forms concerning chest radiographs or chest CT [19,24]. Radiomics is the high-throughput for collecting quantitative image features from medical imaging, which makes it possible to extract and apply data in clinical decision support systems to improve detection or enable information about prognosis, and thus build a bridge between imaging and precision medicine [25,26,27].

However, on-site software solutions and associated studies were rare [22,28], even though the application of AI during clinical routine might result in a reduction in workload and improvement of workflow [29]. 

A fast-to-site clinically oriented approach is also required to reduce the additional costs in the healthcare system [23].

During different stages of the pandemic, the focus of imaging shifted. In the beginning, detection and proof of infection were of the greatest importance. Subsequently, the monitoring of lung complications, long-time changes, and virus subtypes gained attention [30]. In this setting, software approaches are demanded to support clinicians by giving dedicated information about the distribution of affected lung lobes and their individual appearance. The aim of this single-center retrospect study is the implementation of a technically feasible *Pneumonia Analysis prototype*. Moreover, the detection and quantification of pneumonia of different etiologies beyond the primarily trained COVID-19 datasets are tested by using CT scans of patients infected with bacterial and fungal pneumonia. Results are compared to the accuracy of the suspected diagnosis of an experienced reader. 

## 2. Material and Methods

### 2.1. Study Population

**Figure 1:**
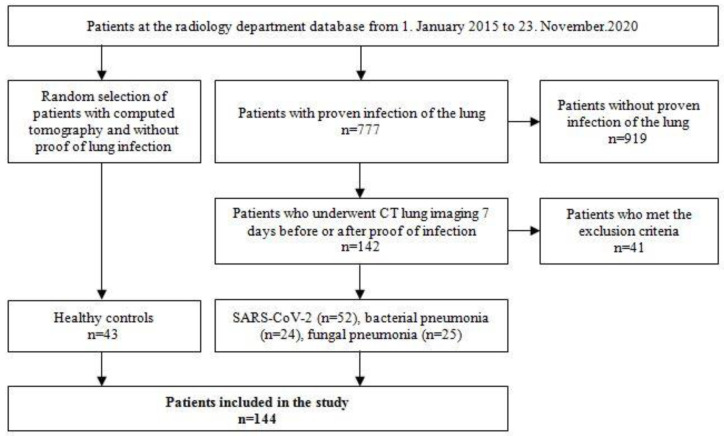
Patient recruitment.

In a single-center retrospective database analysis of the radiological department, 144 patients (58 female, 86 male, mean age 57.7 ± 18.3 years) were identified to be included in the study. There were 52 (36.1%) patients suffering from COVID-19 pneumonia, 24 (16.7%) patients with bacterial, and 25 (17.4%) with fungal pneumonia. A healthy control group of 43 (29.9%) patients was randomly selected from the database of the radiology department. Patient recruitment is shown in Figure 1.

### 2.2. CT Examinations

All CT examinations were performed on state-of-the-art multislice CT scanners (multiple vendors, e.g., SIEMENS, Philips, and TOSHIBA). A 512 × 512 reconstruction matrix, a photon energy of 120 kV, a tube current of 100–150 effective mAs, and a tube rotation time of 0.5 ms was used. The field of view was adjusted for each patient to include the entirety of the chest wall and both lungs. A pitch of 0.6 and a collimation of 128 or 196 × 0.6 mm were used. Intravenous contrast material was applied in 39/144 (27.3%) patients. In all patients, a spiral acquisition was obtained from the apex to the base of the lungs at the end-inspiratory phase. In 8/144 patients, a small part of the basal lung was not recorded. Examinations were performed with patients in the supine position.

### 2.3. Software Technique

**Figure 2:**
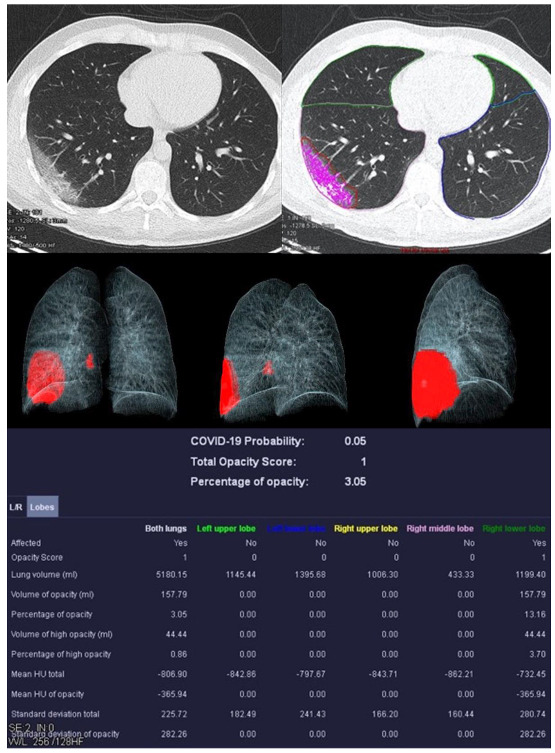
Examination of a 29-year-old male patient with COVID-19 pneumonia. The direct subpleural location of the ground glass opacity in the sense of a pneumonia focus is striking.

**Figure 3:**
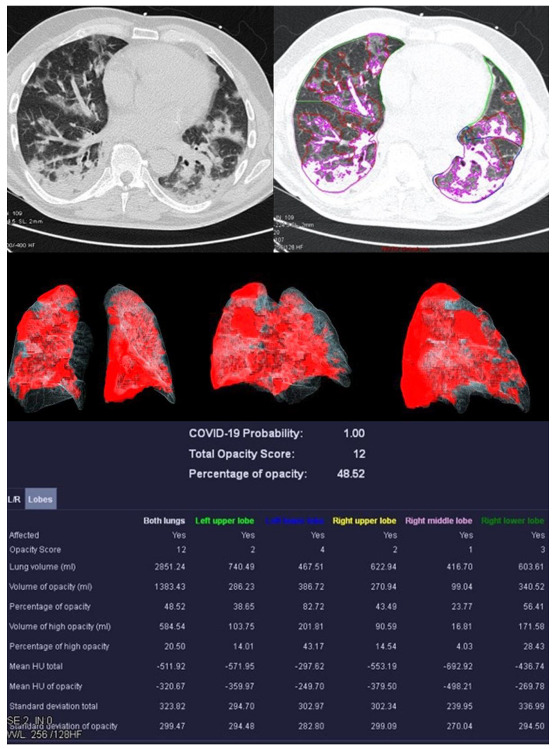
Examination results of a 61-year-old patient with a severe course of COVID-19 pneumonia. Incipiently consolidating ground glass opacities and sharply defined ground glass opacities with reticulations and crazy-paving patterns.

**Figure 4:**
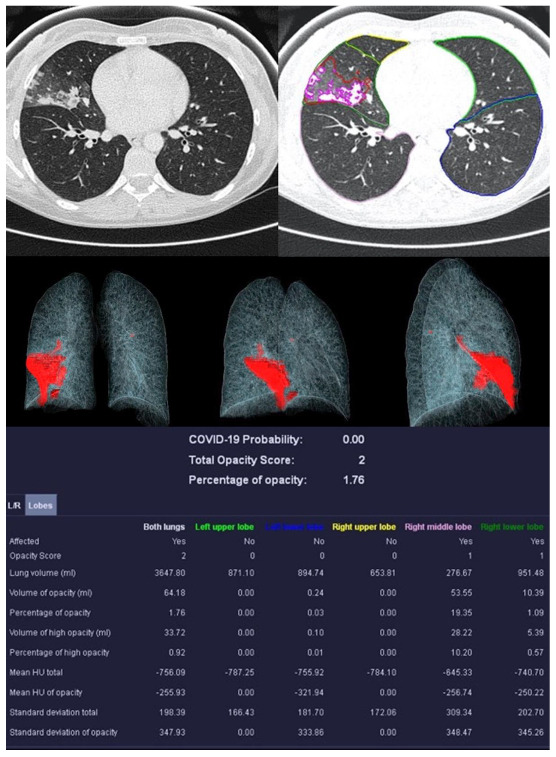
Example examination of a 36-year-old patient with bacterial pneumonia caused by Heamophilus influenzae. There are flat densifications in the middle lobe corresponding to lobar pneumonia.

**Figure 5:**
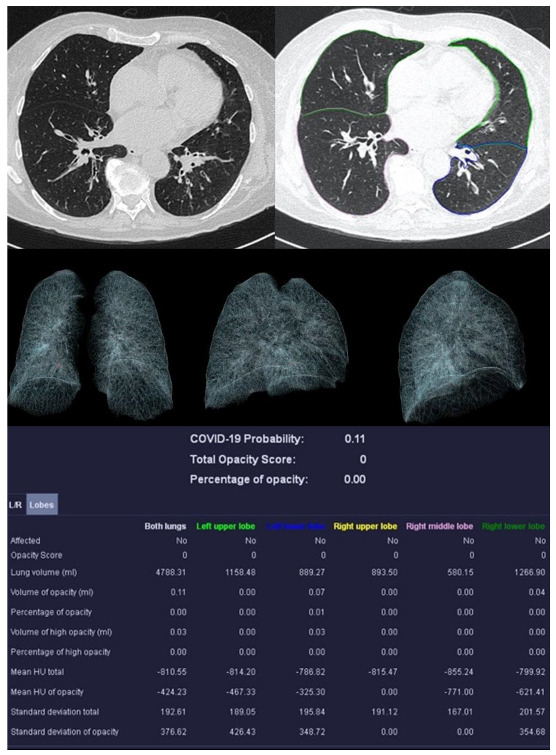
Sample examination of a healthy 67-year-old patient. Only global bronchial wall thickening and mucus impactions can be seen.

Image analysis was performed using *syngo*.via *CT Pneumonia Analysis prototype* (Version 1.0.4.2, Siemens Healthineers, Forchheim, Germany). Figure 2, Figure 3, Figure 4 and Figure 5 show examples of the *syngo*.via prototype output.

The algorithm automatically delineates airspace opacities using a convolutional neural network trained with data that had been manually labeled by clinical experts. 

The network was trained end-to-end as a classification system using binary cross-entropy and used probabilistic sampling of the training data to adjust for the imbalance in the training dataset labels.

**Figure 6:**
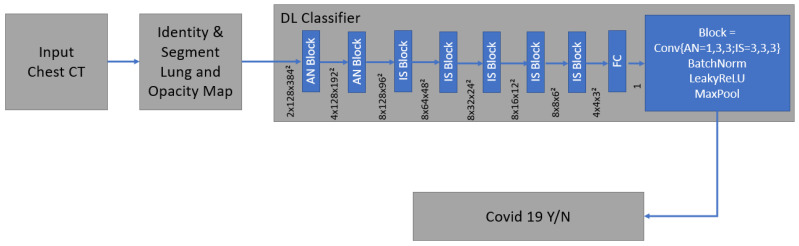
The network was trained end-to-end as a classification system with the binary cross entropy and uses probabilistic samples of the training data. It is supervised deep learning-based COVID-19 classification.

Technical details and development of the algorithm have been described before [28,31]. It is a supervised deep learning-based COVID-19 classification that is explained in detail by Mortani Barbosa EJ et al. [28]. It is a deep learning-based 3D neural network model, M3, that was principally trained to separate the positive class (COVID-19) vs. the negative class (non-COVID-19) (Figure 6) [28].

As an input, we considered a two-channel 3D tensor with the first channel containing directly the CT Hounsfield units within the lung segmentation masks and the second channel containing the probability map of a previously proposed opacity classifier [28,31]. Accordingly, the network uses anisotropic 3D kernels to balance resolution and speed with deep blocks that gradually aggregate function to a binary output. 

The network was trained end-to-end as a classification system with binary cross entropy and used probabilistic samples of the training data to adjust the imbalance in the labels of the training dataset [28]. 

Bernheim et al. reported on the calculation of the opacity score [32]. Two measurements were performed: a first global and a second lobe-specific analysis. The global measurement determines the percentage of opacity (PO), which represents the percentage of all lung tissue affected by COVID-19. The percentage of high opacity (PHO) is the proportion of tissue that is particularly dense (≥−200 HU) and thus represents consolidations.

A lung severity score (LSS) can be generated in the lobe-specific measurement. The proportion of affected tissue is converted into a score of 0–4 for each individual lung lobe. Zero means the lobe is unaffected. At 1, 1–25% is affected, at 2, 26–50%, at 3, 51–75%. With a score of 4, 76–100% of the lobe shows anomalies. The sum of the scores of all five lobes results in the LSS, which can therefore reach values from 0 to 20. The lung high opacity score (LHOS) differs from the LSS in that it only considers areas with a higher density (≥−200 HU).

In addition to the PO, PHO, LSS, and LHOS described above, the quantitative analysis calculates the probability of the presence of COVID-19 pneumonia, called the “COVID-19 Probability”. All volumes (whole lung, individual lung lobes, diseased tissue) are expressed as percentages and in milliliters. Average densities of whole lungs, lobes, and COVID-19 suspect areas are reported in Hounsfield units (HU).

After the automatic analysis is completed, the editing tools are used to adjust the extent of the flagged pneumonia foci to minimize possible segmentation errors by the prototype. Those manual corrections were subsequently verified by an experienced radiologist.

All authors of this paper are end-users of the prototype and do not have access to the development documentation.

### 2.4. Image Analysis

All scans were viewed at standard mediastinal windows (level, 35 HU; width, 450 HU) and lung windows (level, −700 HU; width, 1500 HU). All CT scans were blind evaluated by a dedicated radiologist (BM) with 3 years of experience in thoracic radiology. Probability of COVID-19 pneumonia was classified by using the Coronavirus Disease 2019 Reporting and Data System (CO-RADS) assessments developed by the Dutch Society of Radiology and Prokop et al. [33] in 2020. The suspected pulmonary involvement of possible infection with SARS-CoV-2 in patients with moderate to severe symptoms was classified on a scale of 1 to 5. In addition, the radiologist suggested a clinical diagnosis for each patient, which could conclude COVID-19, bacterial pneumonia, fungal pneumonia, or no pneumonia. 

Furthermore, CT quality was ranked as perfect, good, moderate, or inadequate (PGMI).

### 2.5. Postprocessing Analysis

Additionally, a subgroup classification concerning the criteria of technical postprocessing was performed. Criteria are given in Table 1. 

### 2.6. Microbiological Standard of Reference 

Bacterial infections were proven using sample material collected by bronchoalveolar lavage or sputum for following microscopy. A relevant germ load was ensured by using a number of 1 × 10^5^ or 1 × 10^6^ germs per milliliter as a minimum. Fungal infections were confirmed by positive microscopy or cultured organisms. Sample material was collected from bronchial or tracheal secretion, as well as bronchoalveolar lavage. 

In all patients with COVID-19, nasopharyngeal swabs were collected, followed by RT-PCR assay to confirm the diagnosis. Only patients with a positive RT-PCR result for SARS-CoV-2 were included. Patients within the healthy control group did not receive proof of infection. 

### 2.7. Compliance with Ethical Standards/Ethical Approval

All procedures performed in studies involving human participants were in accordance with the ethical standards of the institutional and national research committee and with the 1964 Helsinki Declaration and its later amendments or comparable ethical standards (No.: 245/20). The study was HIPAA compliant. 

### 2.8. Statistics

Statistical analysis was performed using dedicated software (IBM SPSS 27.0, Armonk, NY, USA). All results are expressed as average and standard deviation. 

The discriminatory ability of the software prototype was evaluated by the area under the receiver operating characteristic (ROC) curve (AUC).

-
$sensitivity=\frac{True\;Positives}{\left(True\;Positives+False\;Negatives\right)}$

-
$specificity=\frac{True\;Negatives}{\left(True\;Negatives+False\;Positives\right)}$

-
$accuracy=\frac{\left(True\;Positives+True\;Negatives\right)}{\left(True\;Positives+True\;Negatives+False\;Positives+False\;Negatives\right)}$

The determination of the specificity in the differentiation of different types of pneumonia by CT Pneumonia Analysis and radiologist was carried out using the chi-squared test and the creation of cross tables. Correlations between metric variables were determined by usage of the Pearson correlation coefficient, between non-metric variables using Spearman’s correlation coefficient. A multivariate ANOVA was used to test the distribution of affected lung tissue for each pathology. A significant difference between the mean values of the affected parts of the lungs was tested using a Games–Howell correction as a post-hoc test. Differences between mean values of COVID-19 probability, lung severity score (LSS), percentage of opacity, and high opacity, as well as mean Hounsfield units, were analyzed using the Kruskal–Wallis test and Dunn–Bonferroni test as a post-hoc test. The influence of the administration of an intravenous contrast medium was calculated using a t-test of two independent samples and a non-parametric U-test.

## 3. Results

### 3.1. Cohort Characteristics

This single-center retrospective analysis consecutively included 144 patients (58 female, mean age 57.72 ± 18.25 years). The mean body mass index (BMI) was 26.62 ± 3.86. The mean height was 171 ± 9 cm, and the mean weight was 69.57 ± 13.18 kg.

### 3.2. Image Analysis

The image quality was distributed from Perfect (*n* = 91, 62.2%) to Good (*n* = 38, 26.4%) and Moderate (*n* = 14, 9.7%) to Inadequate (*n* = 1, 0.7%). The mean postprocessing time was 7.61 ± 4.22 min. A minor correction according to the criteria described above was necessary in 79/144 patients (54.9%), and a major correction in 65/144 patients (45.1%). The use of contrast agents did not influence the results of the software (*p* = 0.81). 

### 3.3. Detection of COVID-19 by Software vs. Reader

**Figure 7:**
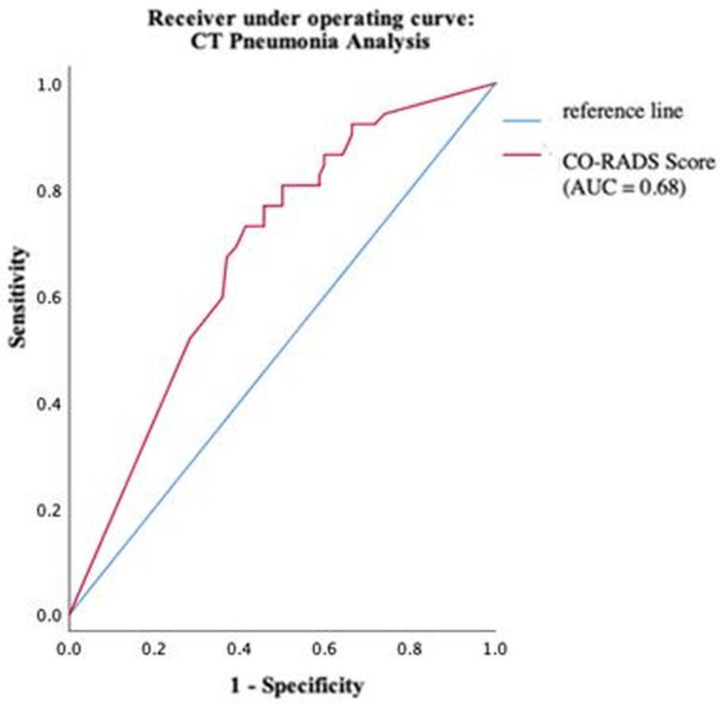
Performance of CT Pneumonia Analysis within different groups of pathogens.

Using ROC analysis, an optimal cut-off value of the COVID-19 probability calculated by CT Pneumonia Analysis was found at 63%. At this cut-off, sensitivity and specificity were maximized. The software reached values of 80.8% and 50%, respectively (Figure 7). 

The human radiologist achieved optimal sensitivity of 80.8% and a specificity of 97.2%. Only 4 patients were wrongly diagnosed with COVID-19 who actually had no pneumonia or another pneumonia type.

The software correctly classified 88/144 patients (61.1%); the radiologist’s diagnosis was correct in 130/144 patients (90.3%), and the difference is statistically significant (*p* < 0.001, φ = 0.34).

Using a logistic regression for the prototype and the radiologist, the radiologist had a significantly stronger predictive power with an odds ratio of 90.91 than the prototype with an odds ratio of 4.20.

**Figure 8:**
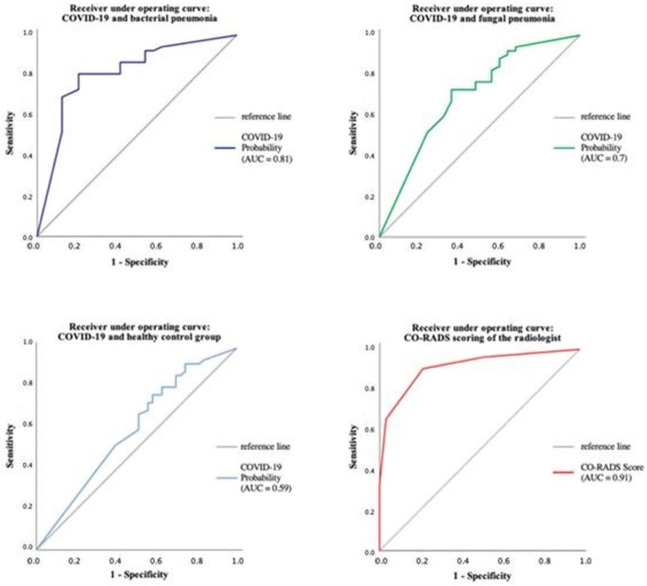
The performance of CT Pneumonia Analysis within the different pneumonia forms using ROC analysis.

The performance of CT Pneumonia Analysis within the different pneumonia forms has also been evaluated using ROC analysis, as can be seen in Figure 8.

### 3.4. Discrimination of Different Pneumonia Types

The radiologist was able to detect 42/52 (80.8%) COVID-19, 18/24 (75%) bacterial, and 8/25 (32%) fungal pneumonia, as can be seen in Table 2. No significant differences between the diagnosis of the subgroups for the reader were registered (*p* = 0.51, φ = 0.12).

In contrast, CT Pneumonia detected 42/52 (80.8%) COVID-19 cases as well (see Table 3). Since the prototype is only testing for COVID-19, no detection rates of other pneumonia can be given.

Patients without pneumonia were detected by the radiologist in 42/43 patients (97.7%) and by software in 16/43 (37.2%) patients. The radiologist was significantly better than the software prototype (*p* ≤ 0.001, φ = 0.65).

Subgroups including confirmed bacterial (*n* = 24, 16.6%), viral (*n* = 52, 36.1%), or fungal (*n* = 25, 16.0%) pneumonia and (*n* = 43, 30.7%) patients without detected pneumonia (comparison group)

### 3.5. Distribution of Different Lung Lobes/Density Distribution

**Figure 9:**
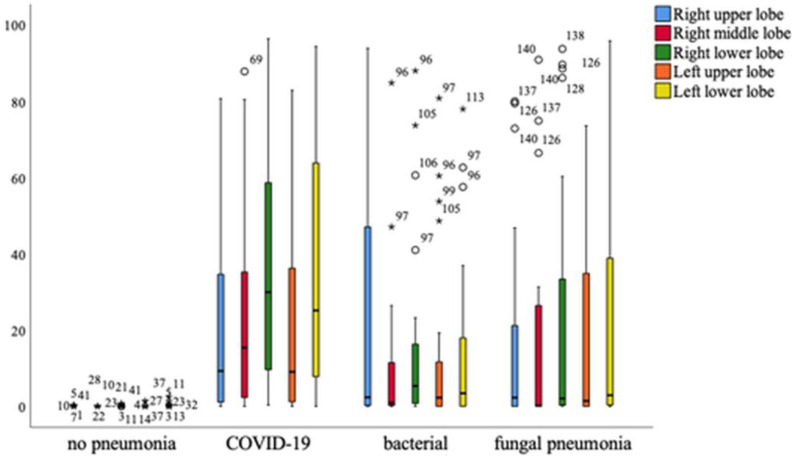
Distribution of different lung lobes of COVID-19 and other pneumonia forms compared to healthy persons. Mild outliers are indicated with a circle, significant outliers with an asterisk. Mild outliers range from 1.5* interquartile range to 3.0* interquartile range to the 3rd quartile. Significant or extreme outliers have a distance of more than 3.0* interquartile range.

Overall, it can be seen that patients with COVID-19 pneumonia show significantly higher levels of affected tissue in all parts of the lung, with the exception of the right upper lobe, which is equally affected in patients with bacterial pneumonia. A significant difference in the affected lung lobes between pathologies was revealed using the one-way multivariant ANOVA, *p* < 0.001 (Figure 9).

Distribution analysis concerning mean COVID-19 Probability, mean LSS/PO/PHO/HU, and HU of opacities were given in Table 4 (global analysis) and Table 5 (lobe-specific analysis).

The mean evaluated COVID-19 probability of 0.80 ± 0.36 is significantly higher in COVID-19 patients than in patients with fungal pneumonia (*p* < 0.05) and bacterial pneumonia (*p* < 0.001). Patients in the healthy control group showed a mean COVID-19 probability of 0.66 ± 0.44 and therefore no statistical difference (*p* = 0.627). Lung severity score (LSS) reflecting the severity of all lung lobes is significantly higher in COVID-19 patients than in the healthy cohort reaching 8 ± 5 (*p* < 0.001). Bacterial and fungal pneumonia show a lower mean of LSS but with no statistical difference to COVID-19 patients (*p* = 0.197, *p* = 0.42). Thereby, mean PO and PHO were significantly higher in COVID-19 than in healthy patients (*p* < 0.001). However, the total mean HU of COVID-19 patients was −679.57 ± 112.72, significantly higher than in healthy patients. The mean HU of the lung in the control group reached −820.18 ± 36.45 (*p* < 0.001). The mean HU of opacity showed no statistical differences since this is already defined as regions of high density (≥−200 HU) within the lung.

### 3.6. Correlation to CO-RADS Classification

Results of CO-RADS classification, CO-RADS score distribution, mean scores, and correlations were given in Supplemental Material.

## 4. Discussion

The precise prediction of COVID-19 pneumonia is of crucial relevance throughout the whole pandemic. The discrimination of COVID-19 and similar pulmonary infections, e.g., other viral infections, especially in high-risk patients (e.g., after bone marrow transplantation) is challenging. Image-based diagnosis using high-resolution chest CT is helpful, but mostly not specific enough [17]. In this study, we aimed at assessing the potential benefit of using the new postprocessing CT Pneumonia Analysis prototype based on chest CT image analysis. COVID-19 detection, as well as differentiation of other pneumonia types, was compared to human reading, performed by a clinical radiologist with three years of experience in thoracic radiology. The prototype algorithm evaluated was designed to automatically identify and quantify abnormal tomographic lung patterns in the context of COVID-19, as well as a black-box approach using an advanced deep learning system [22].

To the best of our knowledge, this is the largest study to evaluate the performance of the CT Pneumonia Analysis prototype on COVID-19 patients, compared to healthy patients and other forms of pneumonia. In addition, only Muñoz-Savreeda et al. (2021) [34] have investigated the usage of a binary classification system based on artificial intelligence on data that cannot be classified as either of the two known groups.

### 4.1. Performance of CT Pneumonia Analysis

Overall, all datasets need minor or major corrections and the mean postprocessing time was high with 7.61 ± 4.22 min compared to other studies using CT Pneumonia Analysis. Gouda and Yasin (2020) [35] reported a manual CT assessment time of as little as 10 s using artificial intelligence. However, Gouda and Yasin did not report manual corrections following the primary analysis of the prototype to match the ground truth of affected lung tissue. Differences in prototype analysis results and ground truth have been stated before by Chaganti et al. (2020).

The possibility of the usage of intravenous contrast agents on data examined with CT Pneumonia Analysis has not been tested before. In this study, it could be shown that contrast-enhancing agents given during the CT examination do not influence the results of the software (*p* = 0.81). This is of large benefit in everyday clinical routines since COVID-19 has been proven to be accompanied by emboli of the lung arteries or thrombosis [36].

### 4.2. Detection of COVID-19 and Differentiation between Other Pneumonia Forms

The software achieved an optimal sensitivity of 80.8% with a specificity of 50%; however, the human radiologist achieved the same sensitivity as the prototype, but was clearly superior in terms of specificity. This means that in summary of all examined patients, the human reader was able to identify the correct diagnosis significantly more often.

Other studies that were aimed to evaluate binary algorithms to differentiate COVID-19 pneumonia and healthy patients while neglecting other etiologies of pneumonia showed promising results as well. Yang et al. (2020) [14], Chen et al. (2020) [37] and Gozes et al. (2020) [15] reported sensitivities for the AI as high as 97%, 97.8% and 98.2%, respectively.

Differentiation of pneumonia caused by SARS-CoV-2 and other pathogens such as the H1N1 influenza virus has been shown by Tabatabaei et al. (2021) [38]. Their system was able to perform with an overall sensitivity of 89% and specificity of 90%. Liang et al. (2022) [39] previously developed *CoviDet*, an AI system that can differentiate between COVID-19 and non-COVID-19 viral infections with a sensitivity and specificity of 98% and 95.6%, respectively. However, all of the named studies used their algorithms on CT datasets of diseases that the artificial intelligence was familiar with, which means that the generalization of the AI was not put to the test. Muñoz-Savreeda et al. (2021) [34] did exactly that by developing a binary deep-learning network that was trained for differentiating COVID-19 and healthy patients on chest X-rays. When using the same algorithm on patients with pneumonia other than COVID-19, 50% of the cases were classified as COVID-19, and 50% as healthy, which resembles a rather random classification.

When it comes to CT Pneumonia Analysis, it was remarkable that especially in the healthy comparison group, the prototype frequently provided the diagnosis of COVID-19. This was not the case with the radiologist who recognized almost all healthy subjects (42/43, 97.7%). The missing one was misinterpreted as a low-marked COVID-19 pneumonia (CO-RADS = 3). In this context, especially areas of insufficiently ventilated lungs were misinterpreted as ground glass.

Homayounieh F. et al. used the same software approach with a maximum AUC of 0.82 which is attributed to differences in patient population, disease severity, comorbidities, and treatment strategies [40]. They discussed that the differences in performance may also be related to significant variations in training and test datasets.

Compared to our study, diagnostic performance was weaker, probably from the somehow more heterogeneous group of different severe forms of COVID-19 pneumonia. Our software may achieve poorer detection in the case of lower and earlier levels of pneumonia, in contrast to a maximum-stage image. The overall small patient cohort can be another partial reason.

Even the sensitivity of chest CT read by a human varies a lot; Xu B. et al. reported in their meta-study about sensitivity in all studies ranging from 0.61 to 0.99 [41]. In this work, the different image quality is also discussed as a cause of different performance, which was not a problem in our work.

Especially, the distinction between COVID-19 and other viral pneumonia findings in the lung has been reported as challenging, with a low specificity of chest CT ranging between 25–33% [11,22,41].

The usefulness of AI-assisted detection could be shown by Song J et al., with an improvement of the average sensitivity of radiologist diagnosis, which was improved from 77 to 85%, and the specificity improved from 75 to 88% [42].

Jia LL et al. showed a pooled area under the curve in their meta-analysis of 32 studies (AUC) 0.96 (95% CI, 0.94–0.98), sensitivity 0.92 (95% CI, 0.88–0.94), pooled specificity 0.91 (95% CI, 0.87–0.93) for the detection of COVID-19 and other pneumonias with a slightly better performance of the AI compared to the human reader. However, they criticized the low methodological quality and the lack of general applicability that is the case with our software approach [43].

Overall, on-site everyday use approaches show somehow lower performance than academically oriented approaches such as in our work [44].

The previously described deficits in the performance of CT Pneumonia Analysis could have several causes: first of all, the prototype was only trained to detect COVID-19 on computed tomography. Even if the presence of bacterial or fungal pneumonia could be evaluated as COVID-19 negative, the algorithm has not “learned” the CT-graphic representation of pneumonia of other etiologies in its training phase, nor in its validation and test phase. Secondly, there are partial overlaps between the morphology of pneumonia of different pathogens [19]. For example, ground glass opacities may represent as the leading change in computed tomography in both COVID-19 and bacterial pneumonia, which falsifies the diagnosis by the CT Pneumonia Analysis prototype, which only knows COVID-19. The investigated algorithm behind the CT Pneumonia Analysis is equivalent to a radiologist in the detection of COVID-19, but inferior in differentiation. The radiologist had a significantly stronger predictive power with an odds ratio of 90.91, than the prototype with an odds ratio of 4.20; however, the radiologist is moderately experienced with 3 years working in thoracic radiology. The radiologist was significantly better than the software prototype (*p* ≤ 0.001, φ = 0.65), especially because the prototype was not trained for detecting other pneumonia forms; however, it was programmed to consider binaries between healthy and COVID-19 patients.

### 4.3. Distribution of Different Lung Lobes

The crucial relevance of the percentage and volume of pulmonary opacities are still consistently superior features for predicting patient outcomes [40,45,46].

As expected, the mean evaluated COVID-19 probability is significantly higher in COVID-19 patients than in other pneumonia subgroups, reaching 0.80 ± 0.36. This is due to the fact that the software was specially trained on COVID-19 pneumonia.

The lung severity score (LSS) reflecting the severity of all lung lobes is 8 ± 5 and significantly higher in COVID-19 than in all healthy patients. This reflects the typically bilaterally appearance of COVID-19 pneumonia with ground glass opacities affecting major parts of each lobe. In contrast to this, mild to moderate cases of fungal and bacterial pneumonia were used because of their characteristic appearance.

Mean PO and PHO were significantly higher in COVID-19 than in healthy patients, reflecting consolidations beside ground glass opacities.

Comparable to Gashi et al. (2021) [22], the major part of our COVID-19 collective has a typical appearance with CO-RADS scores from 3–5; however, not all patients had a CO-RADS 5 score.

### 4.4. Correlation of CO-RADS Classification

Overall, a positive correlation between the CO-RADS score and total opacity score (r = 0.83, *p* < 0.001) was registered, as well as a significant correlation between the percentage of opacity (r = 0.83, *p* < 0.001) and the percentage of high opacity (r = 0.78, *p* < 0.001). Previous studies report similar results. Gashi et al. (2021) [22] quantified affected lung parenchyma using CT Pneumonia as well, showing the correlation between human CO-RADS scoring and total opacity score (r = 0.74), percentage of opacity (r = 0.78), and percentage of high opacity (r = 0.73) [22]. This may be due to the fact that both consolidations and ground glass were accounted for in the CO-RADS classification. In this study, the diagnosis by the radiologist was blinded to the results of the RT-PCR and the detection of germs.

### 4.5. Limitations

There are several limitations to this study. First, the number of patients included was low due to the feasibility nature of the study. Validation in a larger cohort should be aimed as the next step.

Second, only one radiologist was a reader of the datasets. Third, the applied software prototype in this study is not FDA-approved. Fourth, different vendors and CT scanners were used, comparable to a pervious study by Georgescu [28].

## 5. Conclusions

We evaluated the technical feasibility of an AI-based software prototype optimized for the detection of COVID-19 pneumonia in CT datasets of the lung with and without an intravenous contrast agent. The software achieved an optimal sensitivity of 80.8%, equal to a human radiologist; however, with a specificity of 50%, a lower result than a human reader with a specificity of 97.2%. The detection and quantification of pneumonia beyond the primarily trained COVID-19 datasets using the “Pneumonia Analysis prototype” is possible; however, the technical feasibility can be proven.

The advantages of the software approach are the fast, automated segmentation, and quantification of the pneumonia foci, as well as a relatively good transferability to pneumonia of other etiologies. Our study proves the feasibility on site and thus the usability in clinical everyday life using a small cohort. Nevertheless, further studies are necessary to provide a better correlation between clinical information and the information provided by the software. Both must be linked in detail to clinical parameters and the final outcome. Furthermore, in a larger cohort, the differentiation of different forms of pneumonia should be further investigated.

## Figures and Tables

**Table 1:** Subgroup classification concerning criteria of technical postprocessing.

	Major Corrections	Minor Corrections
Postprocessing time	>2 min	<2 min
Removement of artifacts	Pacemaker, stents	Motion artifacts
Correction of pneumonia area	>25%	<25%
Other		– Removal of the segmentation of non-visible changes in the pulmonary framework – Adding/removing airways/blood vessels within a segmentation

**Table 2:** Discrimination of different pneumonia types by radiologist.

	COVID-19	Bacterial Pneumonia	Fungal Pneumonia	No Pneumonia
Sensitivity	80.8%	75%	32%	97.7%
Specificity	95.7%	85.8%	95%	93.1%
PPV	91%	51%	57%	86%
NPV	90%	94%	87%	99%
Accuracy	90%	84%	84%	94%

**Table 3:** Diagnostic performance of CT Pneumonia Analysis on COVID-19.

	CT Pneumonia Analysis on COVID-19
Sensitivity	80.8%
Specificity	50%
PPV	47.8%
NPV	82%
Accurancy	61.1%

**Table 4:** Global analysis of COVID-19 pneumonia compared to other forms using mean COVID-19 probability score and mean density values.

	COVID-19	Bacterial Pneumonia	Fungal Pneumonia	No Pneumonia
Mean COVID-19 Probability ± SD	0.80 ± 0.36	0.33 ± 0.4	0.55 ± 0.47	0.66 ± 0.44
Mean LSS ± SD	8 ± 5	5 ± 4	5 ± 6	0 ± 0
Mean PO ± SD in %	26.39 ± 23.22	12.52 ± 17.97	18.90 ± 26.27	0.05 ± 0.12
Mean PHO ± SD in %	6.42 ± 7.68	3.60 ± 4.47	5.86 ± 10.04	0.01 ± 0.02
Mean HU total	−679.57 ± 112.72	−750.12 ± 84.05	−715.10 ± 37.28	−820.18 ± 36.45
Mean HU of opacity	−453.40 ± 170.46	−427.39 ± 157.92	−450.47 ± 115.38	−416.18 ± 298.62

**Table 5:** Lobe-specific analysis of COVID-19 pneumonia compared to other forms using mean COVID-19 probability score and mean density values.

	COVID-19	Bacterial Pneumonia	Fungal Pneumonia	No Pneumonia
Left upper lobe				
Mean LSS ± SD	1 ± 1	1 ± 1	1 ± 1	0 ± 0
Mean PO ± SD in %	21.80 ± 25.49	13.18 ± 22.97	16.43 ± 22.51	0.07 ± 0.29
Mean PHO ± SD in %	4.07 ± 6.89	5.44 ± 10.97	3.81 ± 6.36	0.00 ± 0.02
Left lower lobe				
Mean LSS ± SD	2 ± 1	1 ± 1	1 ± 2	0 ± 0
Mean PO ± SD in %	35.77 ± 29.92	14.15 ± 22.52	25.15 ± 35.15	0.11 ± 0.42
Mean PHO ± SD in %	10.89 ± 14.57	4.23 ± 8.93	8.74 ± 17.89	0.01 ± 0.02
Right upper lobe				
Mean LSS ± SD	1 ± 1	1 ± 1	1 ± 1	0 ± 0
Mean PO ± SD in %	20.31 ± 22.24	22.79 ± 31.96	17.43 ± 26.20	0.02 ± 0.06
Mean PHO ± SD in %	3.80 ± 5.22	7.30 ± 12.69	4.55 ± 8.04	0.00 ± 0.01
Right middle lobe				
Mean LSS ± SD	1 ± 1	1 ± 1	1 ± 1	0 ± 0
Mean PO ± SD in %	21.73 ± 22.82	10.34 ± 19.70	15.09 ± 25.97	0.00 ± 0.00
Mean PHO ± SD in %	3.30 ± 4.75	2.27 ± 3.99	2.47 ± 4.82	0.00 ± 0.00
Right lower lobe				
Mean LSS ± SD	2 ± 1	1 ± 1	1 ± 1	0 ± 0
Mean PO ± SD in %	37.393 ± 29.669	15.888 ± 24.715	21.819 ± 33.503	0.067 ± 0.173
Mean PHO ± SD in %	11.96 ± 14.31	4.60 ± 10.37	9.19 ± 18.86	0.01 ± 0.06

## Data Availability

Not applicable.

## Supplementary Materials

The following supporting information can be downloaded at: https://www.mdpi.com/article/10.3390/diagnostics13122129/s1, Figure S1: Distribution of CO-RADS scores separated into the different pneumonia forms.; Figure S2: Distribution of mean total opacity score in different CO-RADS subgroups.; Figure S3: Percentage of High Opacity areas distributed to different CO-RADS subgroups/classification.; Figure S4: Percentage of Opacity areas distributed to different CO-RADS subgroups/classification.; Table S1: Mean CO-RADS scores separated into the different pneumonia forms.
