# Supplementary material for: Quantitative Evaluation of COVID-19 Pneumonia CT Using AI Analysis—Feasibility and Differentiation from Other Common Pneumonia Forms
Source: Diagnostics (Basel). 2023 Jun 20;13(12):2129. doi: 10.3390/diagnostics13122129 (PMC10297168; doi:10.3390/diagnostics13122129)
Supplement: Supplementary file 1 [file diagnostics-13-02129-s001.zip › diagnostics-2334905-Supplementarymaterials.pdf]

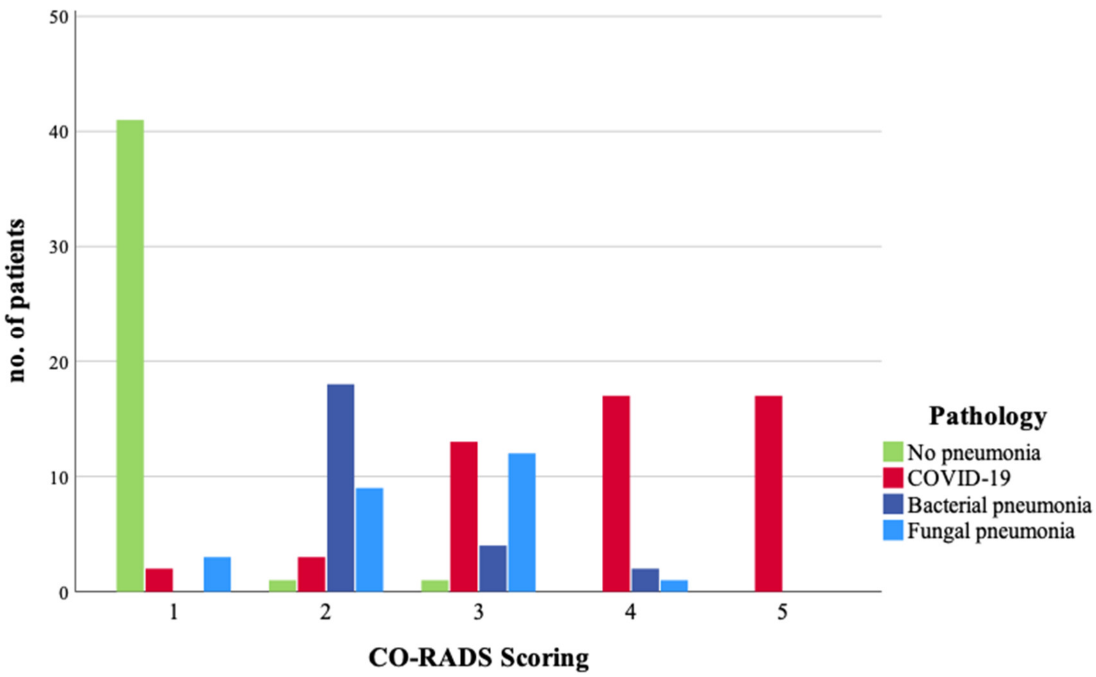

Figure S1. Distribution of CO-RADS scores separated into the different pneumonia forms.

Table S1. Mean CO-RADS scores separated into the different pneumonia forms.

	COVID-19	Bacterial pneumonia	Fungal pneumonia	No pneumonia
Mean CO-RADS Score ± SD	4 ±	2 ± 1	2 ± 1	1 ± 0

While neglecting the individual entities of bacterial, fungal and COVID-19 pneumonia, an overall positive correlation between CO-RADS Score and Total Opacity Score (r = .83, p < 0.001) was registered, as can be seen in the next figure. Furthermore, a significant correlation between Percentage of Opacity (r = .83, p < .001) and Percentage of High Opacity (r = .78, p< 0.001) and CO-RADS classification was present. Including patients with COVID-19 pneumonia only, the correlation between CO-RADS Score and Total Opacity Score were r = .51, p < .001, r = 0.56, p < 0.001 for Percentage of Opacity and r = .5, p < 0.001 for Percentage of High Opacity, respectively. Radiological scoring does not correlate with the value of COVID-19 probability calculated by CT Pneumonia Analysis (spearman correlation coefficient is r = .18, p = 0.16.)

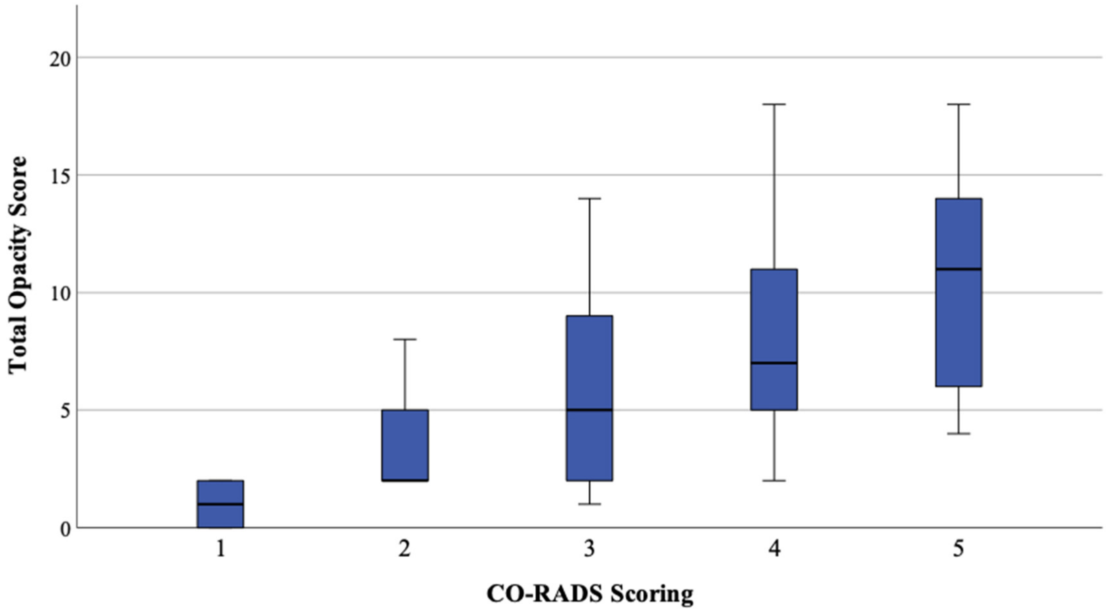

Figure S2. Distribution of mean total opacity score in different CO-RADS subgroups.

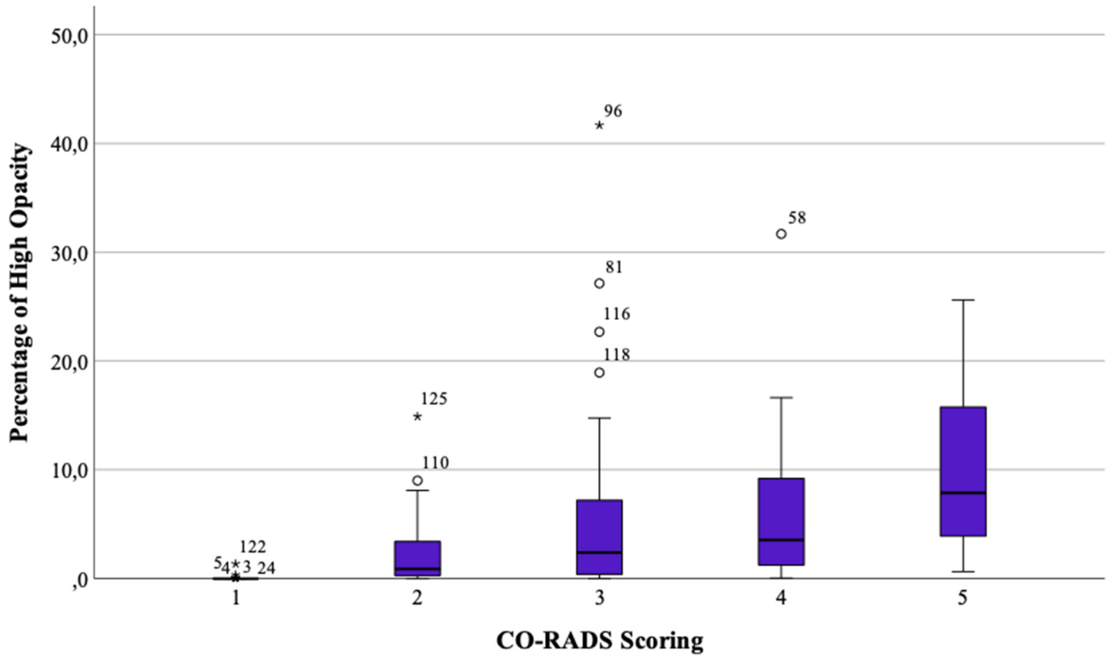

Figure S3. Percentage of High Opacity areas distributed to different CO-RADS subgroups/classification.

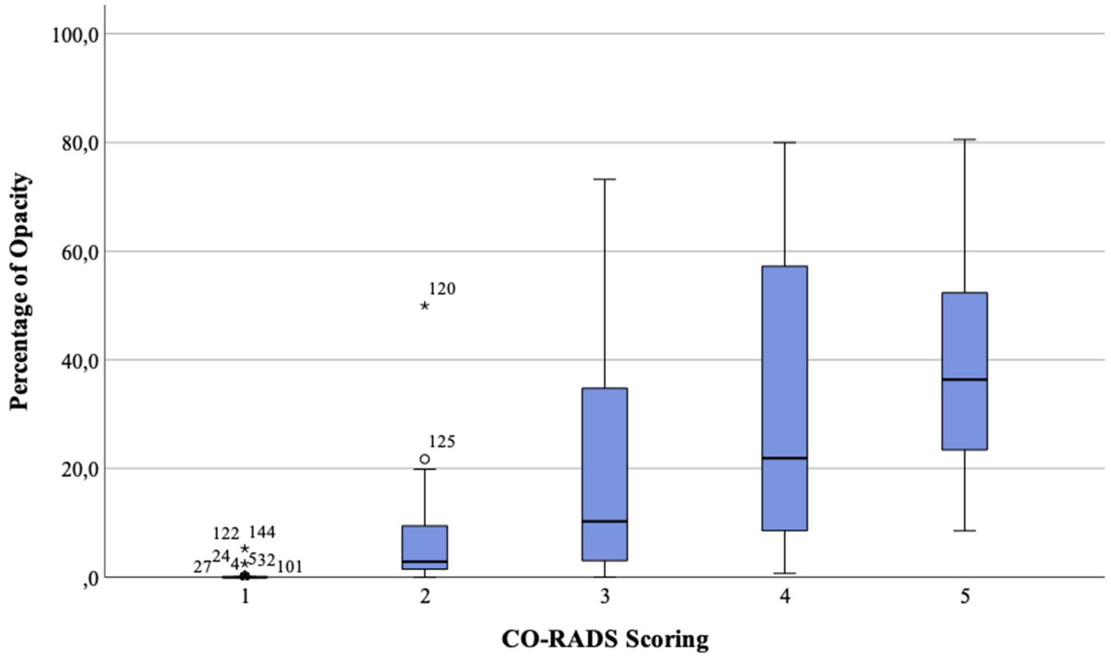

Figure S4. Percentage of Opacity areas distributed to different CO-RADS subgroups/classification.
